# Placenta-derived SOD3 deletion impairs maternal behavior via alterations in FGF/FGFR-prolactin signaling axis

**DOI:** 10.1016/j.celrep.2024.114789

**Published:** 2024-09-25

**Authors:** Yidan Xu, Ana B. Alves-Wagner, Hitoshi Inada, Sepideh D. Firouzjah, Shion Osana, Muhammad Subhan Amir, Royce H. Conlin, Michael F. Hirshman, Eva S. Nozik, Laurie J. Goodyear, Ryoichi Nagatomi, Joji Kusuyama

**Affiliations:** 1 Department of Biosignals and Inheritance, Graduate School of Medical and Dental Sciences, Tokyo Medical and Dental University (TMDU), Tokyo 113-8510, Japan; 2 Department of Medicine and Science in Sports and Exercise, Tohoku University School of Medicine, Sendai 980-8575, Japan; 3 Section on Integrative Physiology and Metabolism, Joslin Diabetes Center, Harvard Medical School, Boston, MA 02215, USA; 4 Department of Developmental Neuroscience, Tohoku University Graduate School of Medicine, Sendai 980-8575, Japan; 5 Division of Biomedical Engineering for Health and Welfare, Graduate School of Biomedical Engineering, Tohoku University, Sendai 980-8575, Japan; 6 Department of Oral and Maxillofacial Surgery, Faculty of Dentistry, Airlangga University, Surabaya 60132, Indonesia; 7 Frontier Research Institute for Interdisciplinary Sciences, Tohoku University, Sendai 980-8578, Japan; 8 Cardiovascular Pulmonary Research Laboratories and Pediatric Critical Care, Department of Pediatrics, the University of Colorado Anschutz Medical Center, Aurora, CO 80045, USA; 9 Lead contact

## Abstract

Offspring growth requires establishing maternal behavior associated with the maternal endocrine profile. Placentae support the adaptations of the mother, producing bioactive molecules that affect maternal organs. We recently reported that placentae produce superoxide dismutase 3 (SOD3) that exerts sustained effects on the offspring liver via epigenetic modifications. Here, we demonstrate that placenta-specific *Sod3* knockout (*Sod3*^−/−^) dams exhibited impaired maternal behavior and decreased prolactin levels. Most fibroblast growth factor (FGF)-regulated pathways were downregulated in the pituitary tissues from *Sod3*^−/−^ dams. FGF1-, FGF2-, and FGF4-induced prolactin expression and signaling via the phosphoinositide 3-kinase (PI3K)-phospholipase C-γ1 (PLCγ1)-protein kinase-Cδ (PKC)δ axis were reduced in primary pituitary cells from *Sod3*^−/−^ dams. Mechanistically, FGF1/FGF receptor (FGFR)2 expressions were inhibited by the suppression of the ten-eleven translocation (TET)/isocitrate dehydrogenase (IDH)/α-ketoglutarate pathway and DNA demethylation levels at the zinc finger and BTB domain containing 18 (ZBTB18)-targeted promoters of Fgf1/Fgfr2. Importantly, offspring from *Sod3*^−/−^ dams also showed impaired nurturing behavior to their grandoffspring. Collectively, placenta-derived SOD3 promotes maternal behavior via epigenetic programming of the FGF/FGFR-prolactin axis.

## In brief

Kusuyama et al. define a mechanism for placenta-derived SOD3 to cultivate maternal behavior. Placenta-specific Sod3 knockout dams exhibited impaired maternal behavior and decreased prolactin levels. SOD3 promotes prolactin expression through the activation of an αKG/IDH/TET axis, resulting in epigenetic changes to FGF/FGFR signals in the pituitary tissue of the dam.

## Graphical Abstract

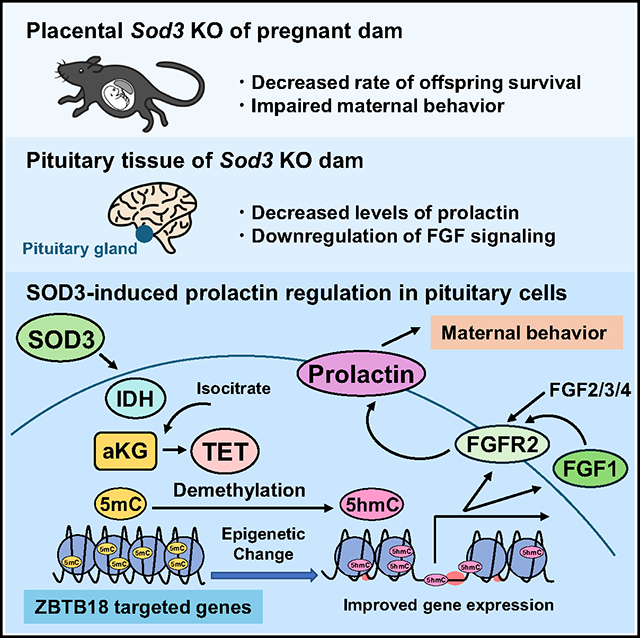

## INTRODUCTION

To support the needs of offspring, pregnancy is characterized by significant changes in the neurological function of the mother.^1^ Proper nurturing of the mother-child bond is crucial in the early stages of the life cycle. It has been reported that at least 10%–15% of women in industrialized countries experience postpartum anxiety and depression, often accompanied by difficulties in child care.^2^ One clinical study found that women who experience anxiety or depression during the postpartum period are often already anxious or depressed during pregnancy.^3^ Several epidemiological and case-control studies have also reported the effects of maternal stress-induced poor caregiving on offspring brain development and the risks of emerging behavioral and mental health problems later in life.^2,4^ These observations suggest that the neurological changes occurring during pregnancy may have adverse effects on the mother’s future nurturing behavior and the healthy development of her offspring.

Immediately after delivery, mothers show significant interest in their pups and exhibit maternal behavior, such as providing food, warmth, shelter, and protection to their pups. The onset of maternal behavior is associated with brain signaling via pregnancy-related endocrine hormones released from intra- or extra-neuronal systems into circulation.^1,5–7^ Placenta is a fetal organ that supports fetal growth via nutrient supplementation, gas exchange, and waste excretion. In addition to fetal development, placentae support the physiological adaptations of the mother that are necessary for successful pregnancy and parenting. Placentae produce steroid hormones and neuroactive hormones that affect numerous maternal organs. For example, placental leptin regulates adipose tissue and hypothalamus, which in turn controls adiposity and food intake during pregnancy.^8^ Importantly, placentae actively produce estrogen,^9,10^ progesterone,^9,11^ and placental lactogen^12–14^ to control pregnancy- and fetus-development-related events. We hypothesize that these multi-faceted placental secretory factors stimulate pup-induced maternal and postpartum nurturing behavior in the dams.

Placental secretion of bioactive molecules is regulated by not only the developmental stage of the placenta but also the exposure of the mother to various stimuli during pregnancy. We previously demonstrated that maternal exercise induces the expression and production of superoxide dismutase 3 (SOD3), also known as extracellular SOD (EC-SOD), in the placenta.^15^ SOD3 is an important transmitter that mediates the benefits of maternal exercise on glucose metabolism in the offspring. Mechanistically, SOD3 induces epigenetic reprogramming, including DNA demethylation and histone H3K4me3 stabilization in the fetal liver,^15,16^ and the effects of the placenta-derived SOD3 exposure on offspring metabolism are sustained after the delivery and expulsion of the placenta. These findings suggest that the placenta reprograms organ function by epigenetic modification after delivery. However, the roles of placental SOD3 on other maternal functions, including behavior during pregnancy, are not known.

Increased physical activity of mothers during pregnancy has long been reported to have positive effects, ranging from reducing the risk of gestational weight gain and diabetes to the decreased rate of postpartum depression.^17^ Physical activity during pregnancy is highly effective in reducing the odds and severity of prenatal depression and anxiety.^18^ The mechanism for these important effects of maternal exercise on the mother’s well-being and nurturing behavior is still unknown. Given the numerous effects of SOD3 on epigenetic regulation and our finding of SOD3 as an exercise-induced placental protein, we hypothesized that SOD3 mediates the beneficial effects of maternal exercise on maternal behavior.

In this study, we used a mouse model of placental *Sod3* depletion and found impaired maternal behaviors including nesting, retrieving, and crouching. Poor maternal behavior was associated with decreased prolactin levels in the serum and pituitary tissues of dams. Placental *Sod3* knockout (KO) inhibited fibroblast growth factor (FGF) signaling-induced prolactin expression through the downregulation of DNA demethylation at the promoters of FGF1 and FGF receptor (FGFR)2. Moreover, placental *Sod3* KO led to poor maternal behavior of offspring toward grandoffspring. This transgenerational effect of placental SOD3 on maternal behavior highlights the importance of the placenta as a secretory organ for both the mother and offspring.

## RESULTS

### Full inhibition of placental SOD3 secretion into maternal blood induces maternal demotivation in dams

**Figure 1.**
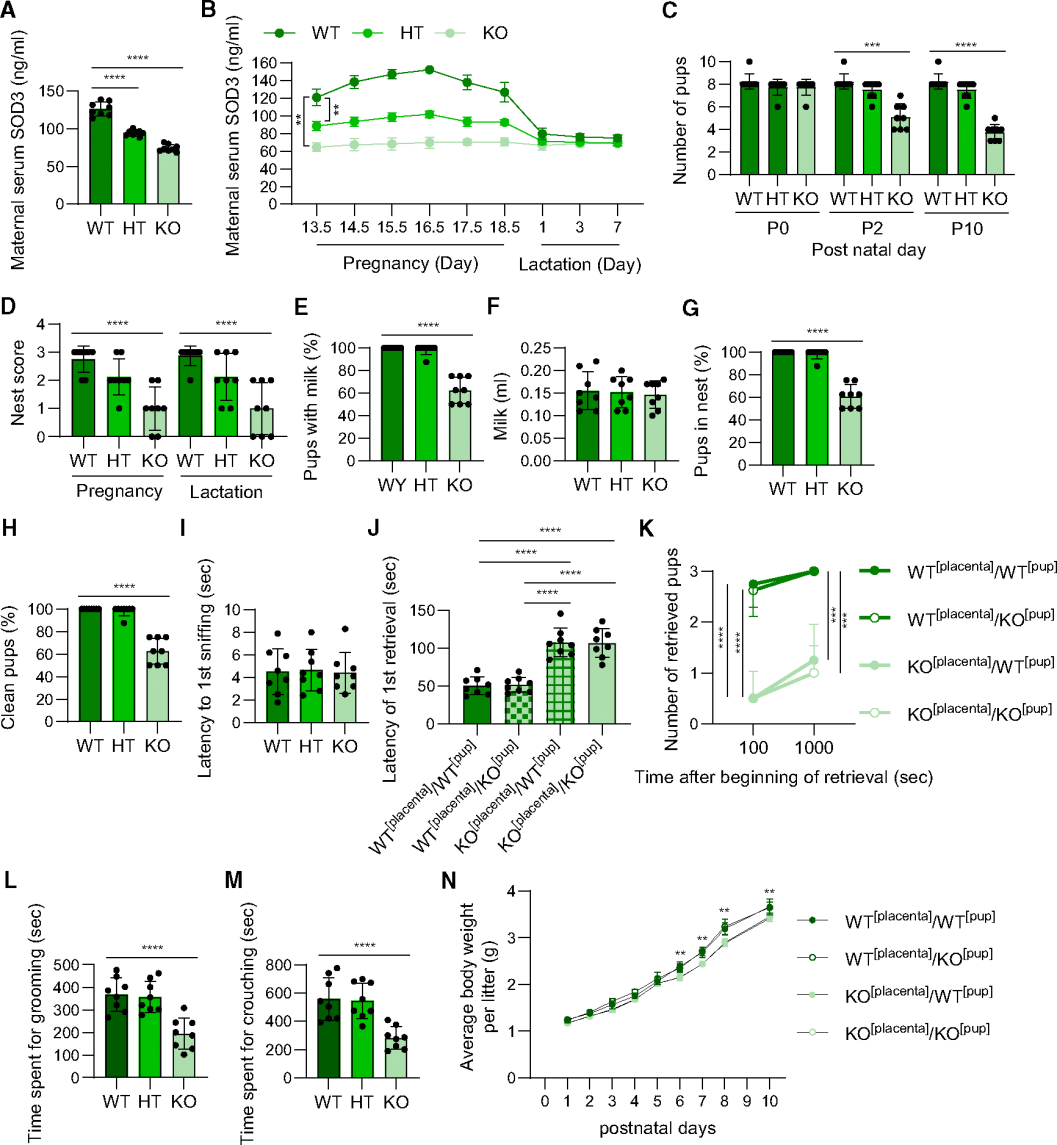
Placental *Sod3* KO has detrimental effects on maternal behavior (A and B) Serum levels of superoxide dismutase 3 (SOD3) in maternal blood on day 18.5 of pregnancy (A) or from day 13.5 of pregnancy to day 7 of lactation (B). Wild type (WT): all placentae are *Sod3*^*f/f*^ in dams; hetero (HT): 50% of placentae are *Sod3*^*f/f*^ and 50% of placentae are *Sod3*^−/−^ in dams; knockout (KO): all placentae are *Sod3*^−/−^ in dams. (C, E–G, and H) Number of living pups (C), rate of pups with milk on day 1 (E), amount of milk on day 1 (F), rate of pups in the nest on day 1 (G), and rate of clean pups on day 1 (H) from WT, HT, and KO dams. (D and I) Nest score at pregnancy and lactation (D) and the first sniffing latency (I) on day 1 of WT, HT, and KO dams. (J) Latency of the first retrieval in the combination of *Sod3*^*f/f*^ and *Sod3*^−/−^ dam and pup on day 1. WT^[placenta]^: dams of placental *Sod3*^*f/f*^; KO^[placenta]^: dams of placental *Sod3*^−/−^; WT^[pup]^: pups from placental *Sod3*^*f/f*^ dams; KO^[pup]^: pups from placental *Sod3*^−/−^ dams. (K) Number of pups retrieved after 100 or 1,000 s in the combination of *Sod3*^*f/f*^ and *Sod3*^−/−^ dam and pup on day 1. (L and M) Time spent grooming (L) and crouching by WT, HT, and KO dams on day 1. (N) Average body weight per litter of postnatal offspring from the combination of *Sod3*^*f/f*^ and *Sod3*^−/−^ dam and pup, respectively. *N* = 8 in each group; three technical replicates for each group; **p < 0.01, ***p < 0.001, and ****p < 0.0001.

To determine the effects of depleting placenta-derived SOD3 on dam nurturing behaviors, we mated *Sod3*^f/f^ female mice with (1) trophoblast-specific protein α (Tpbpa)/adenosine deaminase (Ada) *Cre*^+/−^; *Sod3*^*f/f*^ or (2) Tpbpa/Ada *Cre*^+/+^; *Sod3*^*f/f*^ male mice to generate three types of dams: wild type (WT; all placentae in the dam are *Sod3*^*f/f*^), hetero (HT; 50% of placentae in the dam are *Sod3*^*f/f*^ and 50% of placentae are *Sod3*^−/−^), and KO (all placentae in the dams are *Sod3*^−/−^ in dams). As expected, we found that serum levels of SOD3 in the maternal blood on day 18.5 of pregnancy were significantly decreased by 25.3% in HT dams and 42.2% in KO dams (Figure 1A). The effects of placental Sod3 KO on the decreases of maternal serum SOD3 levels started from day 13.5 to 18.5 of pregnancy; however, SOD3 levels were not changed after delivery or during the lactation period (Figure 1B). There were no differences among genotypes in the female/male ratio (WT: 51% ± 11% female, HT: 55% ± 16% female, KO: 53% ± 17% female). However, as the pups aged during early life, there were profound effects of altered maternal SOD3 levels by placental *Sod3* KO on dam nurturing and behavioral parameters and pup survival. While the number of live pups at delivery (postnatal day [P]0) was normal, the survival rate of pups from KO dams gradually declined from P0 to P10 (Figure 1C). Nest-building quality was significantly worsened in placental Sod3 KO dams during both pregnancy and the lactation period (Figure 1D). The ratio of breastfed pups (Figure 1E), the amount of secreted milk (Figure 1F), pups in the nest (representing nurturing behavior by dams; Figure 1G), and clean pups (representing licking behavior by dams; Figure 1H) were similarly decreased. We then performed a retrieval assay for the same genotype of dam and pup pairs. The latency of the first sniffing, showing the immediate social response of dams directed toward pups, was not different among the genotypes (Figure 1I). To examine whether the detrimental effects of placental Sod3 KO on dam nurturing behavior are caused by the pup or dam genotypes, we used a combination of *Sod3*^*f/f*^ and *Sod3*^−/−^ dams and pups and performed retrieval assays in four experimental groups: (1) WT^[placenta]^/WT^[pup]^: dam of placental *Sod3*^*f/f*^ and pups from placental *Sod3*^*f/f*^ dams, (2) WT^[placenta]^/KO^[pup]^: dam of placental *Sod3*^*f/f*^ and pups from placental *Sod3*^−/−^ dams, (3) KO^[placenta]^/WT^[pup]^: dam of placental *Sod3*^−/−^ and pups from placental *Sod3*^*f/f*^ dams, and (4) KO^[placenta]^/KO^[pup]^: dam of placental *Sod3*^−/−^ and pup from placental *Sod3*^−/−^ dams (Figure 1J). The dam’s retrieval of the first pup back to the nest was not dependent on the pup genotypes, as there was no difference in retrieval time between WT^[pup]^ and KO^[pup]^. In contrast, there was a clear effect due to the lack of SOD3 in the placenta, as KO^[placenta]^ took 2.2 times longer than WT^[placenta]^ for the first retrieval regardless of pup genotypes. WT^[placenta]^ returned almost all WT^[pup]^ and KO^[pup]^ to their nest within 100 s, whereas after allowing for a retrieval time of 1,000 s, dams with KO^[placenta]^ only retrieved a maximum of one pup (Figure 1K). KO dams exhibited less time performing maternal behaviors, including grooming (Figure 1L) and crouching (Figure 1M), compared to WT or HT dams. These poor maternal behaviors of KO^[placenta]^ dams were associated with the impaired growth of pups from P6 to P10 (Figure 1N). Collectively, these results indicate that normal SOD3 expression in placenta is necessary for normal maternal behavior.

### Placental *Sod3* KO-induced decreases in prolactin levels result in poor maternal behavior in dams

**Figure 2.**
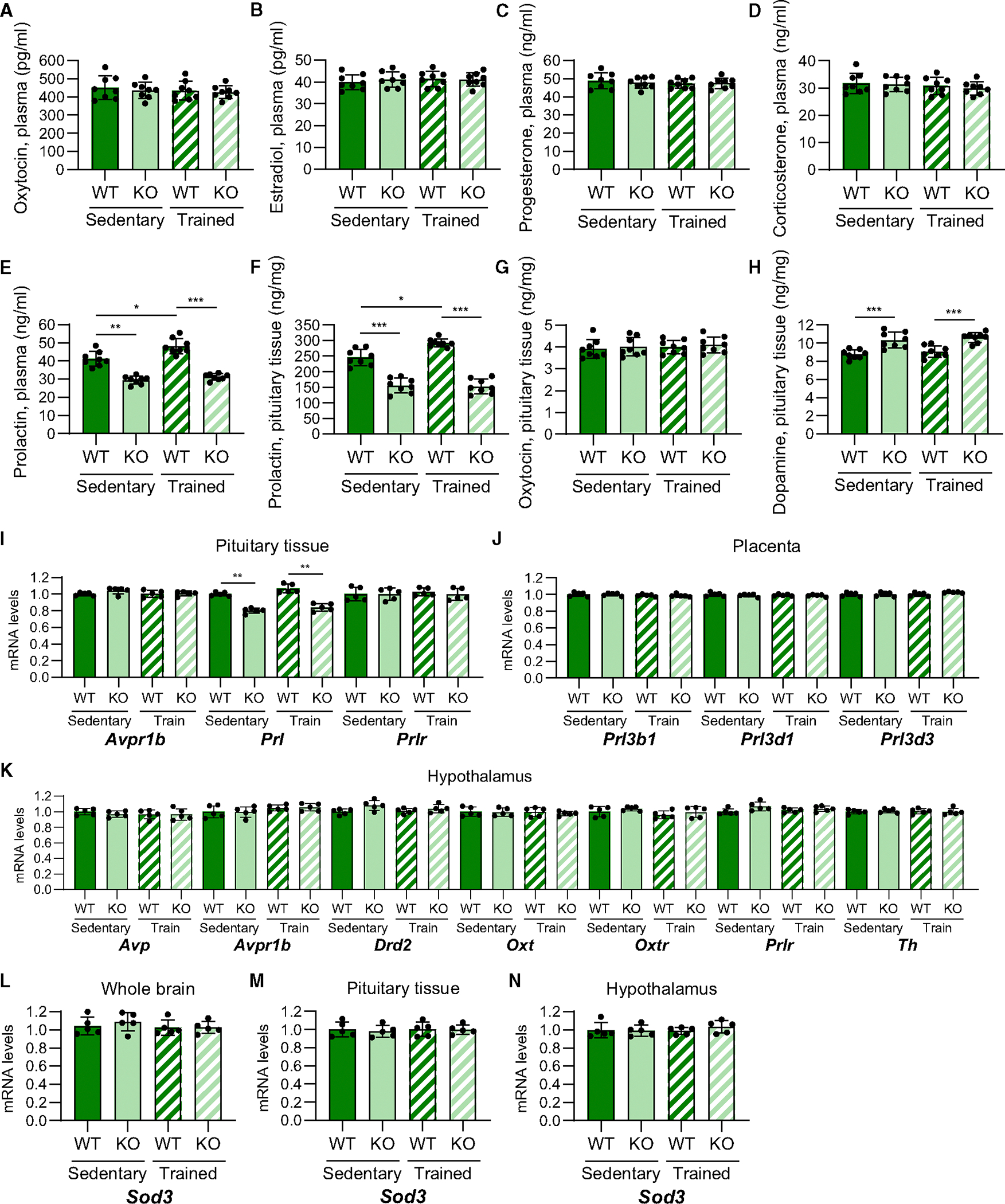
Placental *Sod3* KO decreases prolactin levels in serum and pituitary tissues (A–E) Plasma levels of prolactin (A), oxytocin (B), estradiol (C), progesterone (D), and corticosterone (E) in sedentary or trained, WT or KO dams on day 18.5 of pregnancy. (F–H) Levels of prolactin (F), oxytocin (G), and dopamine (H) in the pituitary tissue of sedentary or trained, WT or KO dams on day 18.5 of pregnancy. (I–K) Maternal behavior-related gene expression in the pituitary gland (I), placenta (J), and hypothalamus (K) from sedentary or trained, WT or KO dams on day 18.5 of pregnancy. (L–N) The mRNA expression levels of Sod3 in the whole brain, pituitary tissue, and hypothalamus of sedentary or trained, WT or KO dams on day 18.5 of pregnancy. (A–J) *N* = 8 in each group. (K–N) *N* = 5 in each group. Three technical replicates for each group (*p < 0.05, **p < 0.01, and ***p < 0.001).

Levels of neuroendocrine hormones, female sex hormones, and steroid hormones during pregnancy control maternal behavior after delivery.^1,5–7^ Considering that we found that placental SOD3 regulates maternal behavior, we analyzed maternal behavior-related hormones in the plasma of sedentary and trained, WT and KO dams on day 18.5 of pregnancy. Plasma levels of oxytocin (Figure 2A), estradiol (Figure 2B), progesterone (Figure 2C), and corticosterone (Figure 2D) were unaffected by maternal exercise and placental Sod3 KO. In contrast, a lack of placental SOD3 resulted in a striking change in prolactin levels (Figure 2E). In the baseline sedentary state, plasma prolactin concentrations were significantly lower in placental *Sod3* KO dams compared to WT dams. Training increased prolactin concentrations in WT dams; however, the effects of maternal exercise were completely blocked in KO dams. Sedentary placental *Sod3* KO dams also had lower levels of prolactin in their pituitary tissue, and training increased the pituitary prolactin concentration in WT, but not KO, dams (Figure 2F). Pituitary oxytocin levels were not changed by training or placental Sod3 KO (Figure 2G). KO dams had significantly higher dopamine levels in pituitary tissues, whereas maternal exercise did not affect the dopamine levels in either WT or KO dams (Figure 2H). The expression and secretion of prolactin are regulated by the hormonal feedback/forward system in the pituitary gland.^19^ We then examined the gene expression of hormones, hormone receptors, and hormone metabolic enzymes that regulate prolactin secretion in pituitary tissue, placenta, and hypothalamus (Figures 2I–2K). Following the prolactin concentrations in plasma and pituitary tissue of KO dams, mRNA expression of *prolactin* (*Prl*) was suppressed in the pituitary gland of sedentary and trained KO dams (Figure 2I). In contrast, the gene expression of other neuroendocrine hormones (*arginine vasopressin* [*Avp*], *oxytocin* [*Oxt*]), placental prolactin families (*Prl3b1*, *Prl3d1*, *Prl3d3*), hormone receptors (*arginine vasopressin receptor 1B* [*Avpr1b*], *prolactin receptor* [*Prlr*], *dopamine receptor D2* [*Drd2*], *oxytocin receptor* [*Oxtr*]), and *tyrosine hydroxylase* (*Th*) were not affected by maternal exercise and placental *Sod3* depletion in pituitary tissue, placenta, and hypothalamus of dams. We confirmed that mRNA levels of *Sod3* in the whole brain (Figure 2L), the pituitary tissue (Figure 2M), and the hypothalamus (Figure 2N) were not affected by placental Sod3 depletion. Collectively, placental Sod3 KO decreases the serum and pituitary tissue levels of prolactin in the dams.

**Figure 3.**
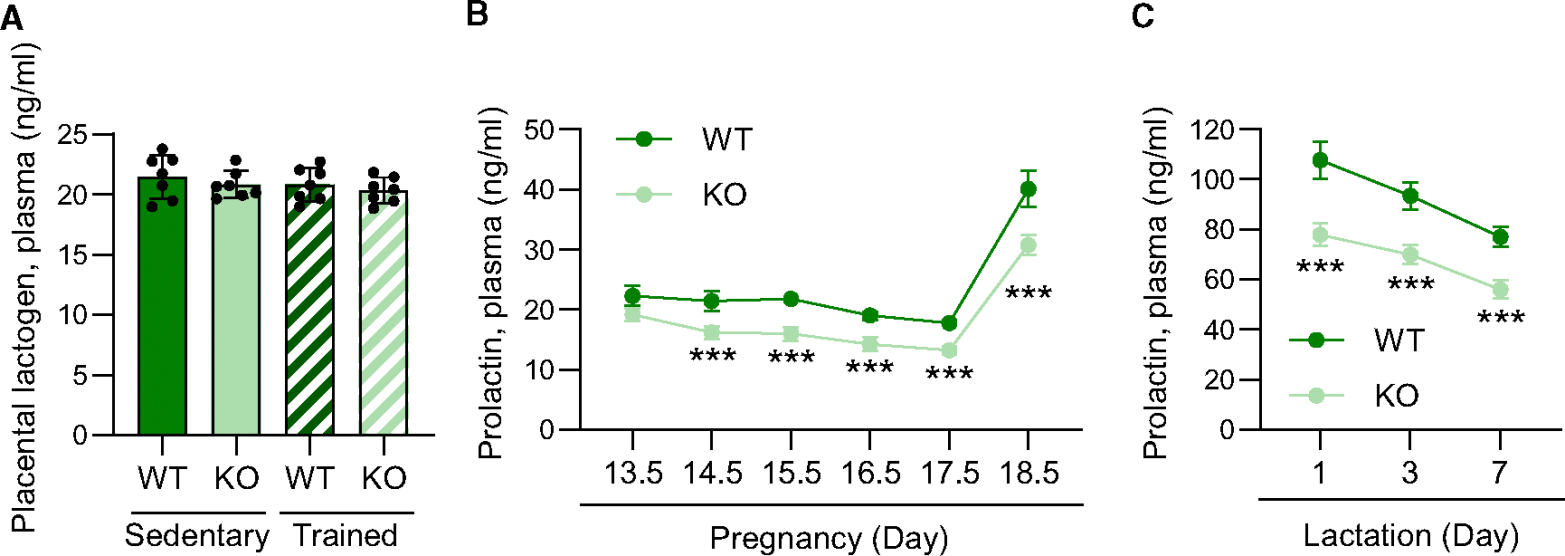
Placental *Sod3* KO suppresses prolactin levels during pregnancy and lactation (A) Plasma levels of placental lactogen in sedentary or trained, WT or KO dams on day 18.5 of pregnancy. (B and C) Plasma levels of prolactin during pregnancy (B) and lactation (C) period in WT or KO dams. *N* = 8 in each group; three technical replicates for each group; ***p < 0.001.

Furthermore, we analyzed the dynamics of prolactin secretion during pregnancy and lactational period. Placental lactogen is known as the main ligand to the prolactin receptor in late pregnancy.^20^ Plasma placental lactogen levels were not changed by training or placental Sod3 KO (Figure 3A). We then analyzed the plasma levels of prolactin at multiple time points from during pregnancy in WT and placental Sod3 KO dams (Figure 3B). Prolactin levels were continuously suppressed from day 14. 5 to 18.5 in pregnancy by placental SOD3 deletion. Additionally, we analyzed the plasma prolactin levels in early lactation at days 1, 3, and 7 of WT and placental Sod3 KO groups (Figure 3C). The levels of prolactin are suppressed in all three time points in lactation by placental SOD3 deletion.

**Figure 4.**
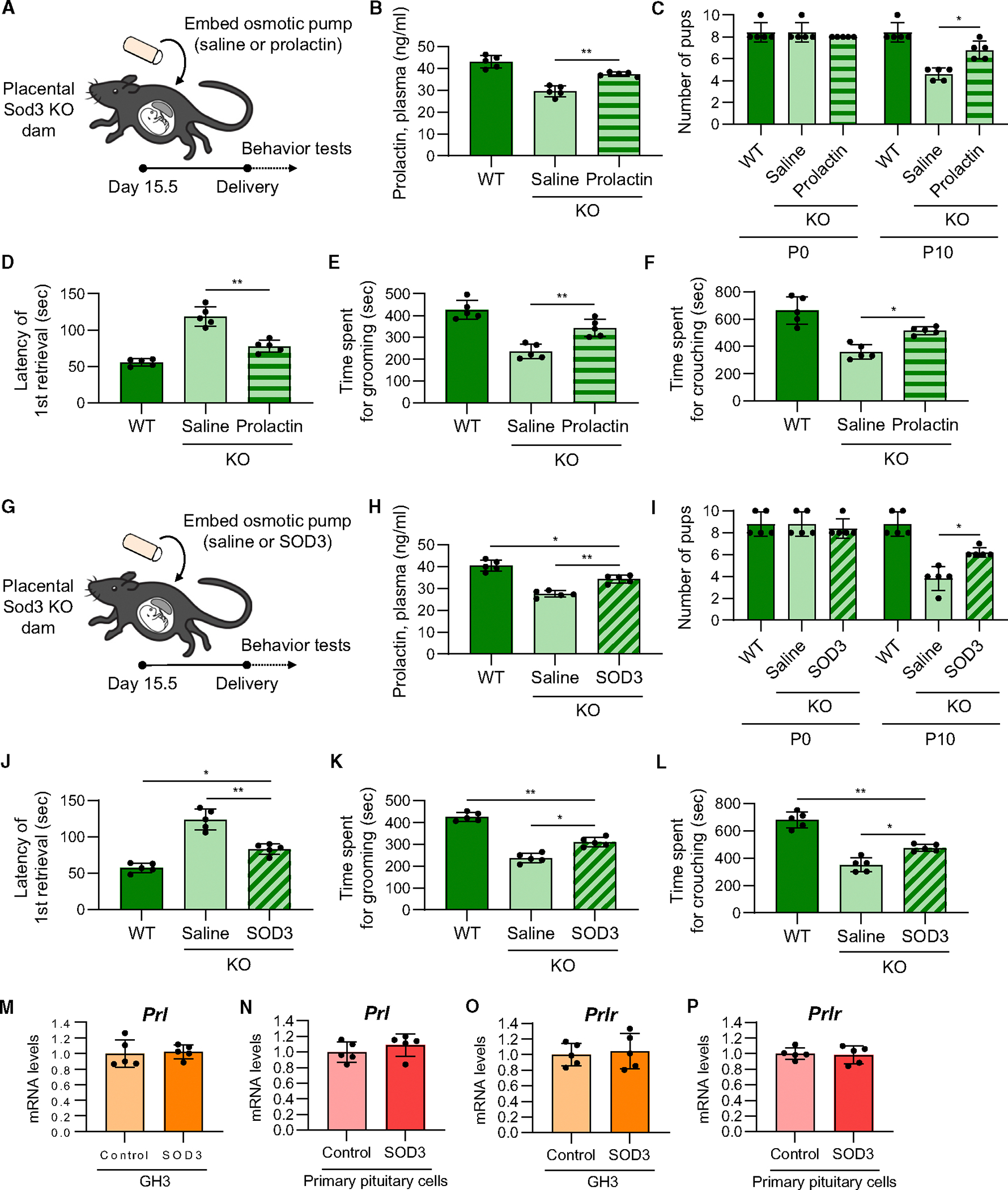
Prolactin and SOD3 supplementation rescue the inhibitory effects of placental *Sod3* deletion on maternal behavior (A and G) Time course of prolactin (A) or SOD3 (G) osmotic pump embedding and maternal behavior tests in placental *Sod3* KO dam. (B–F and H–L) Plasma levels of prolactin (B and H), number of living pups (C and I), latency of the first retrieval on day 1 (D and J), time spent grooming on day 1 (E and K), and time spent crouching on day 1 (F and L) of WT dams and KO dams with prolactin (B–F) or SOD3 (H–L) osmotic pump embedding of saline or recombinant prolactin. (M–P) mRNA expression levels of *prolactin* (*Prl*) (M and N) and *prolactin receptor* (*Prlr*) (O and P) in 200 ng/mL recombinant SOD3-stimulated GH3 cells (M and O) and mouse primary pituitary cells (N and P). *N* = 5 in each group; three technical replicates for each group; *p < 0.05 and **p < 0.01.

To examine the involvement of placental SOD3 in prolactin-induced maternal behavior, *Sod3* KO dams were infused with recombinant prolactin (Figures 4A–4F), recombinant SOD3 (Figures 4G–4L), or saline via an osmotic pump from day 15.5 to 20.5 of pregnancy, a time point of the increased production of placental SOD3^15^ and pituitary prolactin^21^ during pregnancy, and maternal behavior tests were performed after delivery. Infusion of prolactin or SOD3 increased plasma prolactin concentrations in the KO dams near normal levels (Figures 4B and 4H). Prolactin or SOD3 supplementation did not affect the number of pups; however, the survival of pups at P10 from KO dams supplemented with prolactin was partially increased compared to that of KO dams with saline (Figures 4C and 4I). Similarly, the detrimental effects of *Sod3* KO on the rates of retrieval (Figures 4D and 4J), grooming (Figures 4E and 4K), and crouching (Figures 4F and 4L) were reversed by prolactin or SOD3 treatment.

Furthermore, we injected 0.1 mg/kg/day of cabergoline, an inhibitor of prolactin secretion, into pregnant mice from day 12.5 to 18.5 of pregnancy. Daily cabergoline injection significantly suppressed plasma levels of prolactin during pregnancy (Figure S1A). Cabergoline treatment had detrimental effects on the latency of retrieval, time spent grooming, and time spent crouching (Figures S1B–S1D).

Based on these *in vivo* results, we hypothesized that SOD3 directly promotes the expression of prolactin or prolactin receptor in pituitary cells. We stimulated GH3, a rat pituitary cell line, or mouse primary pituitary cells from pregnant dams with 200 ng/mL recombinant SOD3 for 24 h. SOD3 treatment did not affect *Prl* and *Prlr* mRNA expression levels in these cells (Figures 4G–4J). Collectively, these results suggest that placenta-derived SOD3 regulates prolactin production and maternal behavior in pregnant dams but does not directly induce prolactin expression in pregnant dams.

### Placental *Sod3* KO-induced prolactin expression is caused by epigenetic inhibition of FGFR signaling

**Figure 5.**
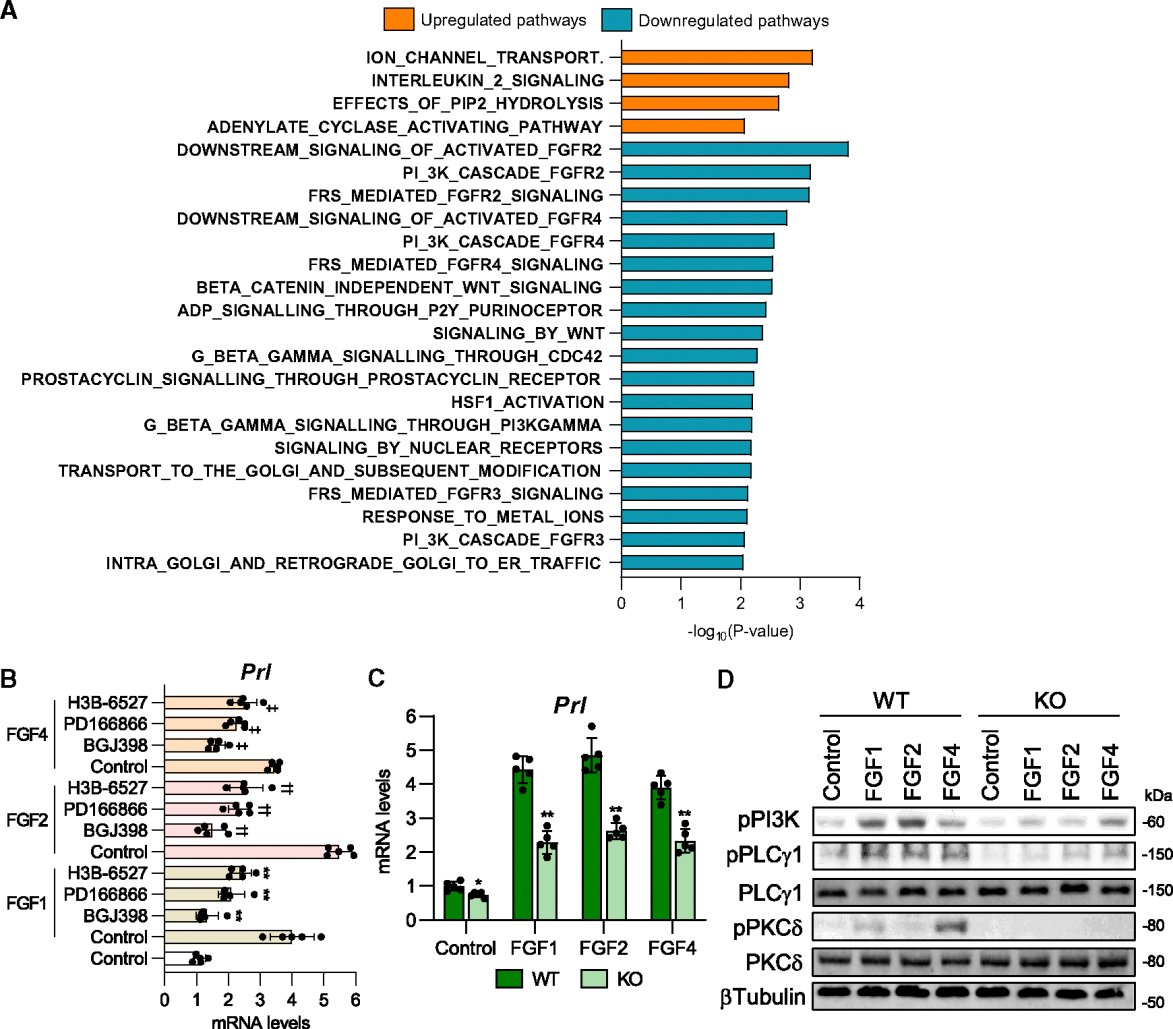
Placental *Sod3* KO inhibits the FGF-prolactin axis in the pituitary gland (A) Reactome pathway analysis of placental *Sod3*^*f/f*^ and *Sod3*^−/−^ dams. (B) *Prl* mRNA expression levels of 100 ng/mL recombinant fibroblast growth factor (FGF)1-, FGF2-, or FGF4-stimulated mouse primary pituitary cells with or without 10 μM FGF receptor (FGFR) inhibitors. BGJ398: FGFR1/2/3 inhibitor, PD16686: FGFR1 inhibitor, H3B-6527: FGFR4 inhibitor. *N* = 5; three technical replicates for each group; **p < 0.01 vs. FGF1-control, ††*p* < 0.01 vs. FGF2-control, and ‡‡*p* < 0.01 vs. FGF4-control. (C) *Prl* mRNA expression levels in recombinant FGF1-, FGF2-, or FGF4-stimulated primary pituitary cells from placental *Sod3*^*f/f*^ and *Sod3*^−/−^ dams on day 10 after delivery. (D) Phosphorylation levels of FGFR-induced signaling molecules in recombinant FGF1-, FGF2-, or FGF4-stimulated primary pituitary cells from placental *Sod3*^*f/f*^ and *Sod3*^−/−^ dams. *N* = 3 in each group; three biological and technical replicates for each group.

To explore the molecular mechanisms by which placental SOD3 regulates prolactin expression in the pituitary tissue, we performed RNA sequencing (RNA-seq) of the pituitary tissues in WT and KO dams and found 4 significantly upregulated pathways and 19 downregulated pathways in placental *Sod3* KO pituitary tissues (Figure 5A). Of note, 7 FGF and FGFR signal-related pathways (DOWNSTREAM_SIGNALING_OF_ACTIVATED_FGFR2, PI_3K_CASCADE_FGFR2, FRS_MEDIATED_FGFR2_SIGNALING, DOWNSTREAM_SIGNALING_OF_ACTIVATED_FGFR4, PI_3K_CASCADE_FGFR4, FRS_MEDIATED_FGFR3_SIGNALING, and PI_3K_CASCADE_FGFR3) were ranked in the top 19 downregulated pathways. Previous studies have reported that FGF1, FGF2, and FGF4 positively regulate prolactin expression and secretion *in vitro* and *in vivo*,^22–26^ suggesting a potential relationship between SOD3 levels and FGF/FGFR signaling. We stimulated mouse primary pituitary cells from pregnant WT dams with 100 ng/mL recombinant FGF1, FGF2, or FGF4 and 10 μM FGFR inhibitors, including BGJ398 (FGFR1, FGFR2, and FGFR3 inhibitor), PD166866 (FGFR1 inhibitor), and H3B-6527 (FGFR4 inhibitor) (Figure 5B). All three FGF treatments promoted prolactin mRNA expression in pituitary cells, and all four FGFR blockers attenuated FGF1-, FGF2-, and FGF4-induced prolactin expression. We also found that the pregnant KO dam-derived pituitary cells had lower responses to FGF1-, FGF2-, and FGF4-induced prolactin expression than WT dam-derived cells (Figure 5C). Similarly, the phosphorylation levels of FGF-prolactin axis-regulating signaling molecules phosphoinositide 3-kinase (PI3K), phospholipase C-γ1 (PLCγ1), and protein kinase-Cδ (PKCδ)^25,27^ were attenuated in KO-derived cells under FGF1, −2, or −4 stimulations (Figure 5D). These results indicate that FGF-FGFR signal-induced prolactin expression is downregulated in pituitary cells of placental *Sod3* KO dams.

**Figure 6.**
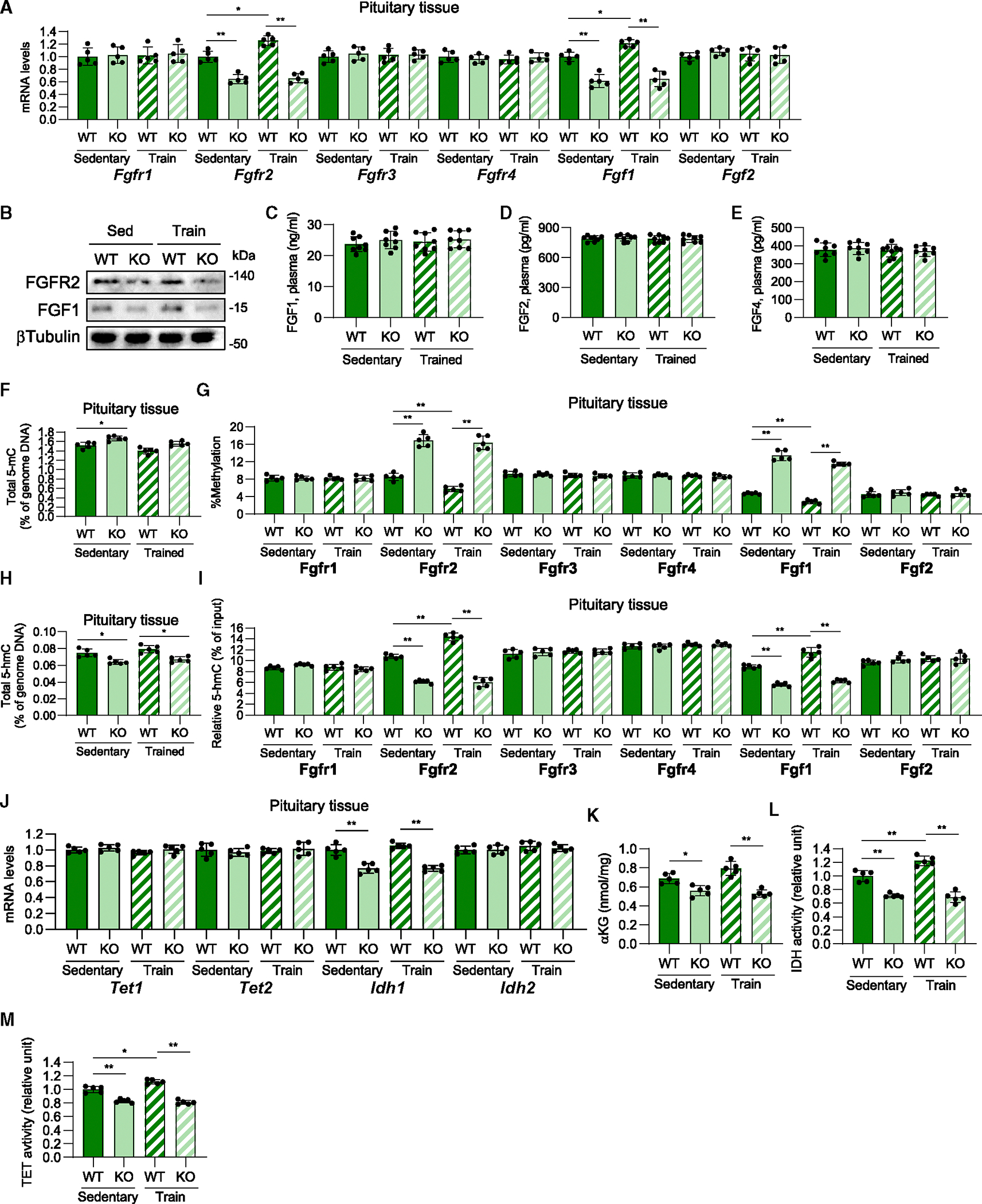
Placental *Sod3* KO suppresses FGFR2 and FGF1 expression via increased promoter DNA methylation in the pituitary gland. Pituitary glands were collected at day 18.5 during pregnancy (A) Gene expression levels of FGFRs and FGFs of the pituitary gland in sedentary or trained, WT or KO dams. (B) FGFR2 and FGF1 protein expression levels in the pituitary gland from sedentary or trained, WT or KO dams. (C–E) Plasma levels of FGF1 (C), FGF2 (D), and FGF4 (E) in sedentary or trained, WT or KO dams. (F and H) Total 5-mC (F) and 5-hmC (H) of pituitary tissues in sedentary or trained, WT or KO dams. (G and I) Relative DNA methylation levels (G) and 5-hmC abundance (I) at the promoter of Fgfr/Fgf genes in the pituitary gland from sedentary or trained, WT or KO dams. (J) Gene expression levels of *Tet* and *Idh* of the pituitary gland in sedentary or trained, WT or KO dams. (K–M) Levels of α-ketoglutarate (αKG) (K) and enzymatic activity of IDH (L) and TET (M) in the pituitary glands of sedentary or trained, WT or KO dams. *N* = 5 in each group; three technical replicates for each group. For (B), three biological replicates for each group. *p < 0.05 and **p < 0.01.

To examine the cause of FGF/FGFR downregulation by placental *Sod3* depletion, we analyzed the expression levels of FGFs and FGFRs in pituitary tissues of placental *Sod3* KO dams on day 18.5 of pregnancy. Among FGFR and FGF families, we found that the mRNA (Figure 6A) and protein (Figure 6B) expression levels of FGFR2 and FGF1 were significantly decreased in the pituitary tissue of KO dams. Exercise during pregnancy increased Fgfr2 and Fgf1 gene expression, whereas these effects were blocked in KO dams. In contrast, the plasma levels of FGF1 (Figure 6C), FGF2 (Figure 6D), and FGF4 (Figure 6E) did not differ between sedentary or trained WT or KO dams. Since our previous study demonstrated that placental SOD3 increases the expression of liver metabolic genes through DNA demethylation, which is caused by the conversion of 5-methylcytosine (5-mC) to 5-hydroxymethylcytosine (5-hmC),^28^ at the promoters of these genes,^15^ we analyzed the levels of 5-mC and 5-hmC in genome DNAs and the Fgfr2 and Fgf1 promoters in the pituitary tissues of placental *Sod3* KO. Total 5-hmC levels of pituitary tissues were significantly increased by placental *Sod3* KO (Figure 6F). 5-mC levels of the top apparent CpG island in the −1,000 upstream region of Fgfr2 and Fgf1 genes were significantly increased in KO dam pituitary tissues relative to WT controls and were not decreased by exercise during pregnancy (Figure 6G). In accordance with the increased 5-mC levels, 5-hmC DNA immunoprecipitation qPCR showed that 5-hmC levels at pituitary tissues (Figure 6H) and the same promoter regions of Fgfr2 and Fgf1 were decreased by placental *Sod3* KO in dam pituitary tissues and were not decreased by training (Figure 6I). Similarly, the DNA methylation levels were not changed at the promoter of Fgfr1, Fgfr3, Fgfr4, of Fgf2. DNA demethylation is mediated by ten-eleven translocation (Tet) enzymes, and the Tet enzymatic reaction is upregulated by α-ketoglutarate (αKG) and isocitrate dehydrogenase 1 (Idh1) and Idh2, enzymes involved in αKG production.^28^ We found that *Idh1* mRNA expression was suppressed in the pituitary glands of *Sod3* KO dams (Figure 6J). Correspondingly, the level of αKG (Figure 6K), IDH activity (Figure 6L), and TET activity (Figure 6M) were downregulated in the pituitary glands of *Sod3* KO dams. Exercise during pregnancy did not affect *Tet* and *Idh* expression; however, IDH and TET activities were upregulated. These results indicate that placental *Sod3* KO attenuates FGFR2 and FGF1 expression through TET-IDH inactivation-induced increases in DNA methylation levels at the promoter of Fgfr2 and Fgf1 genes.

**Figure 7.**
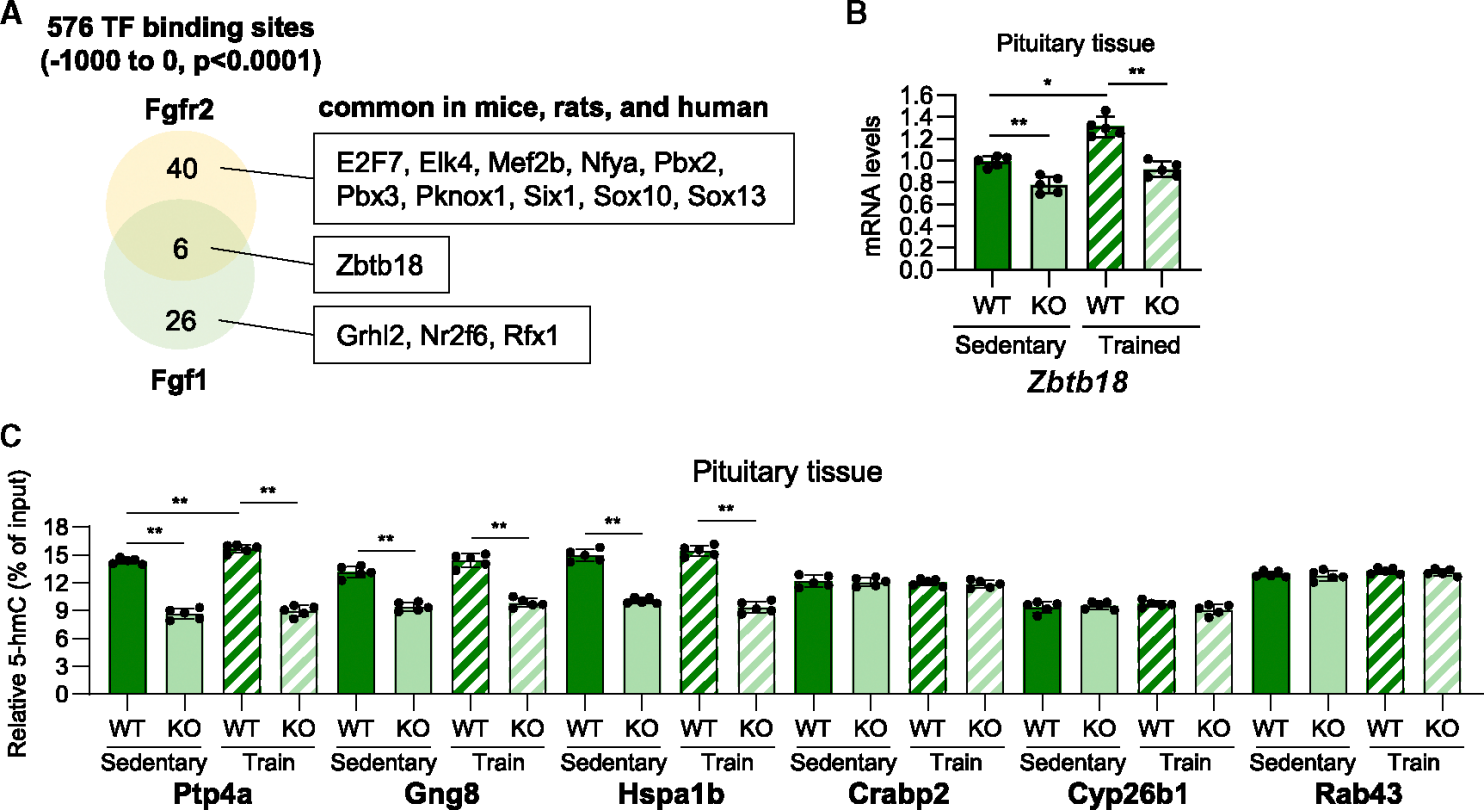
Placental *Sod3* KO suppresses prolactin levels during pregnancy and lactation (A) Potential binding sites of transcription factors (TFs) at the promoters of Fgfr2 and Fgf1 in mice, rats, and humans. (B) Gene expression levels of *Zbtb18* in sedentary or trained, WT or KO dams on day 18.5 of pregnancy. (C) 5-hmC abundance at the promoter of Ptp4a, Gng8, Hspa1b, Crabp2, Cyp26b1, and Rab43 genes in the pituitary tissues from sedentary or trained, WT or KO dams on day 18.5 of pregnancy. *N* = 5 in each group; three technical replicates for each group; *p < 0.05 and **p < 0.01.

Previous studies reported that DNA demethylations at specific target genes are induced by the coupling of the transcription factors with TET enzymes.^29^ We searched the binding sites of the putative transcription factors at Fgfr2 and Fgf1 promoter regions (−1,000 to 0) by JASPER software (Figure 7A). We found 40 transcription factors of Fgfr2 and 26 transcription factors of Fgf1. 11 transcription factor (E2F7, Elk4, Mef2b, Nyfa, Pbx2, Pbx3, Pknox1, Six1, Sox10, Sox13, Zbtb18) binding sites at the Fgfr2 promoter and 4 transcription factor (Grhl2, Nr2f6, Rfx1, Zbtb18) binding sites at the Fgf1 promoter were commonly found in mice, rats, and humans. Of these, zinc finger and BTB domain containing 18 (ZBTB18) was the only transcription factor that could bind to Fgfr2 and Fgf1 promoters. We found that mRNA expression levels of Zbtb18 were significantly upregulated in the trained pregnant mice of WT and Sod3 KO (Figure 7B). Next, to analyze the effects of placental Sod3 KO on DNA methylation of the other genes in pituitary tissues, we picked up protein tyrosine phosphatase 4A1 (Ptp4a), guanine nucleotide-binding protein gamma 8 (Gng8), and heat shock protein family A member 1B (Hspa1b), which are in the bottom 30 downregulated genes in Sod3 KO with potential ZBTB18 binding sites and apparent CpG islands at their promoter regions. We analyzed the DNA demethylation levels of these genes and found that placental Sod3 KO significantly decreased the amount of 5-hmC at these promoter regions (Figure 7C). In contrast, placental Sod3 KO did not affect the DNA demethylation levels of cellular retinoic acid-binding protein 2 (Crabp2), cytochrome P450 26B1 (Cyp26b1), or Rab43 (RAB43; member RAS oncogene family), which are in the bottom 30 downregulated genes in Sod3 KO without potential ZBTB18 binding sites. These results suggest that ZBTB18 potentially guides TET-induced specific DNA demethylation at the promoters with ZBTB18 binding sites including Fgfr2 and Fgf1.

### Placental *Sod3* deletion affects the maternal behavior of offspring

**Figure 8.**
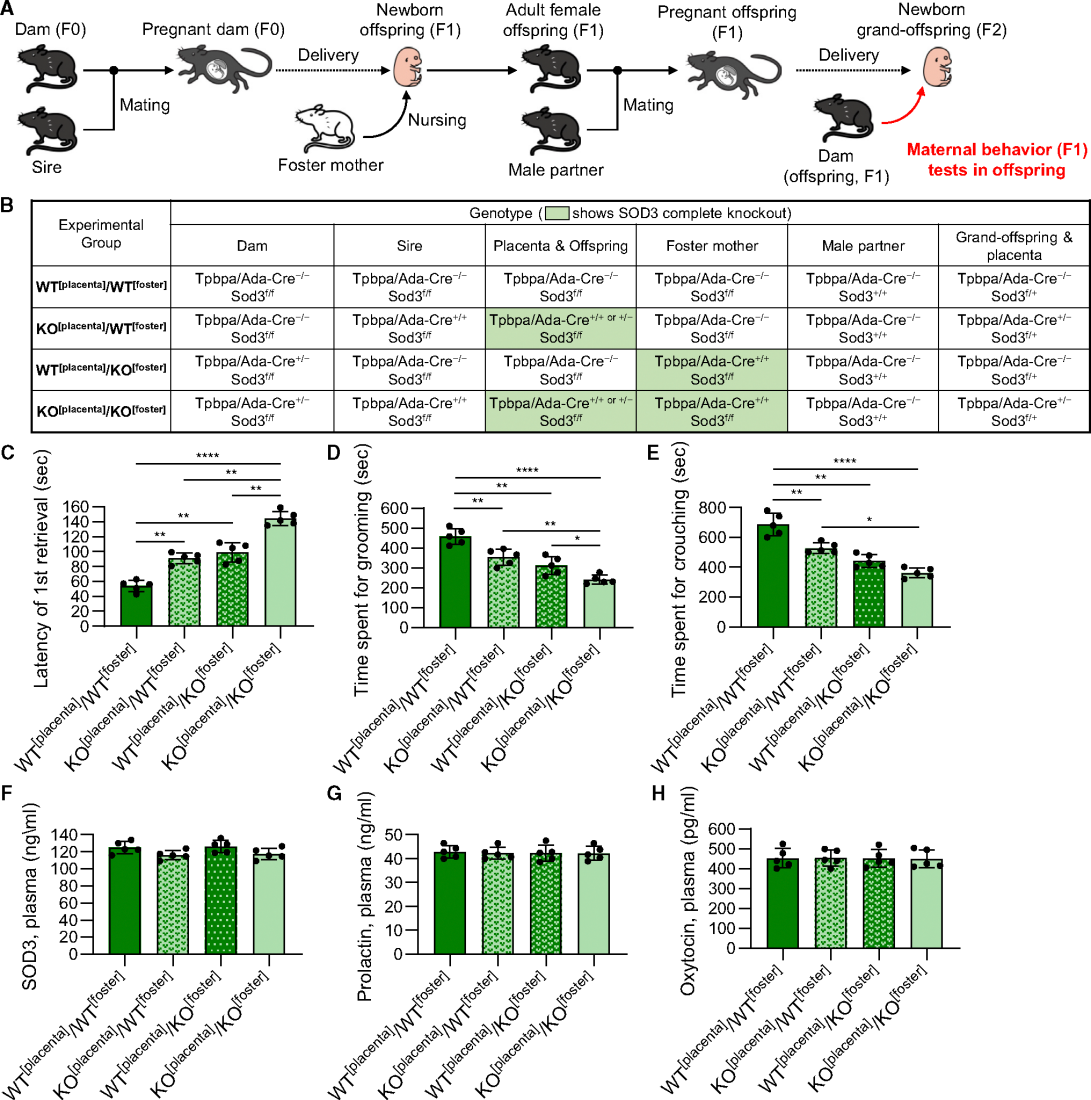
Placental *Sod3* KO in the mother negatively affects the maternal behavior of the offspring (A) Schematic illustration of maternal behavior analysis of the offspring delivered by placental *Sod3*^*f/f*^ and *Sod3*^−/−^ dams and nursed by placental *Sod3*^*f/f*^ and *Sod3*^−/−^ foster mothers. (B) Genotype information of each experimental group. (C–H) Latency of the first retrieval on day 1 (C), time spent grooming on day 1 (D), time spent crouching on day 1 (E), and plasma levels of SOD3 (F), prolactin (G), and oxytocin (H) on day 18.5 of pregnancy in the offspring of each experimental group. *N* = 5 in each group; three technical replicates for each group; *p < 0.05, **p < 0.01, ***p < 0.001, and ****p < 0.0001).

Placenta-derived SOD3 is secreted at both maternal and fetal sites during pregnancy.^15^ Given the effects of placental SOD3 on pituitary tissues and maternal behavior in dams (F0), we hypothesized that placental SOD3 also affects the maternal behavior of offspring (F1). Previous studies have indicated that offspring that have experienced poor nurturing conditions showed poor maternal behavior during their parenting period.^30–32^ To distinguish the effects of SOD3 exposure on fetal offspring (F1) during the developmental period and the effects of poor maternal behavior by the dam (F0) on newborn offspring (F1), four experimental groups—(1) WT^[placenta]^/WT^[foster]^: the placentae in the dam are *Sod3*^*f/f*^ and the placentae in the foster mother are *Sod3*^*f/f*^, (2) KO^[placenta]^/WT^[foster]^: the placentae in the dam are *Sod3*^−/−^ and the placentae in the foster mother are *Sod3*^*f/f*^, (3) WT^[placenta]^/KO^[foster]^: the placentae in the dam are *Sod3*^*f/f*^ and the placenta in the foster mother are *Sod3*^−/−^, and (4) KO^[placenta]^/KO^[foster]^: the placentae in the dam are *Sod3*^−/−^ and the placentae in the foster mother are *Sod3*^−/−^—were set by the genotype combination of the dam (F0), sire, foster mother, and offspring’s (F1) male partner genotypes (Figures 8A and 8B). 8- to 10-week-old females (F0) were mated with 8- to 10-week-old males, the offspring (F1) at embryonic day (E)18.5 were removed from the uterus, and the resuscitated newborns were parented by the foster mother from birth onwards. Then, 8-week-old female offspring (F1) were mated with 8- to 10-week-old males. Behavioral tests of offspring (F1) were performed on P1 after the delivery of grandoffspring (F2). The offspring of KO^[placenta]^/WT^[foster]^ as well as WT^[placenta]^/KO^[foster]^ took 1.8 times longer than WT^[placenta]^/WT^[foster]^ for the first retrieval regardless of pup genotypes (Figure 8C). The offspring of KO^[placenta]^/KO^[foster]^ showed much longer retrieval time than KO^[placenta]^/WT^[foster]^ and WT^[placenta]^/KO^[foster]^. Similarly, the time spent grooming (Figure 8D) and crouching (Figure 8E) was shorter in the offspring of KO^[placenta]^/WT^[foster]^ and WT^[placenta]^/KO^[foster]^ compared to WT^[placenta]^/WT^[foster]^ and much shorter in KO^[placenta]^/KO^[foster]^. We then measured the plasma levels of SOD3 (Figure 8F), prolactin (Figure 8G), and oxytocin (Figure 8H) in the pregnant offspring (F1); however, there were no significant differences among the four groups. These results suggest that both exposure of placental SOD3 to fetal offspring (F1) during the developmental period and poor nurturing experiences by placental Sod3 KO dams (F0) during the offspring’s neonatal period affect the maternal behavior of the offspring (F1). However, poor maternal behavior in offspring (F1) is not related to low levels of serum prolactin.

## DISCUSSION

Physiological and molecular interaction between the mother and their offspring during the pregnancy period is important not only for fetal development but also for the cultivation of motherhood.^33^ Pregnancy-related metabolic changes and motivation for maternal behavior are primarily regulated by endocrine hormones, neuropeptides, and neuro-modulatory systems in the brain.^1,5–7^ However, the roles of the placenta, a pregnancy-limited unique organ for feto-maternal communication during pregnancy, have been underestimated with regard to the interaction between the endocrine system and placenta-derived secretory proteins in the development of maternal behavior. Here, we demonstrated that placental SOD3 promotes maternal behavior through the expression of prolactin in the pituitary tissue. SOD3 induces DNA demethylation at the promoters of Fgf1 and Fgfr2, activating the FGF1/FGFR2-prolactin signaling axis and motivating maternal behavior. Our results indicate that placental SOD3 helps mothers prepare for the multifaceted behavioral and neural changes necessary for parenting.

Maternal prolactin levels during late pregnancy^34^ and prolactin action in the medial preoptic area (MPOA)^35^ are important for generating postpartum maternal nurturing behavior. Prolactin induces the activation of MPOA neural circuitry,^34,35^ the rewarding stimulus to dams,^36^ restraint of aggressive behavior,^37^ and prevention of anxiety-like behavior.^38^ These indispensable roles of prolactin in cultivating maternal behavior partly explain why the only placental *Sod3* KO-induced decreased expression of prolactin showed the comprehensive feature of poor maternal behavior. RNA-seq data of pituitary tissue from placental *Sod3* KO dams extensively changed a variety of differently expressed genes and pathways (Figure 5A), suggesting the possibility of additional regulators of maternal behavior. For example, placental *Sod3* KO induced the upregulation of ADENYLATE_CYCLASE_ACTIVATING_PATHWAY (Figure 5A) and increased the dopamine levels in pituitary tissues (Figure 2H). Considering that dopamine D receptor activation by adenylyl cyclase promotes maternal behavior in rats,^39^ the decreased levels of prolactin may partly compensate for the onset of maternal behavior by modifying dopamine-connected neural circuits. A recent study using prolactin receptor KO mice showed that a reduction in the voluntary running distance in early pregnancy is mediated by prolactin.^40^ On the other hand, we previously showed that voluntary wheel running is not affected in placental *Sod3* KO mice.^15^ These findings suggest that the regulatory effects of prolactin on the motivation of running behavior were different at the stages of pregnancy. Of note, the matured placenta-derived SOD3-prolactin axis seems to be independent of running motivation during the middle-to-late pregnancy period. The other types of neuronal regulators may also modify or rescue nurturing and running motivation.

In mice, prolactin levels show a small elevation in the light/dark phase during early pregnancy and rapidly increase from late pregnancy to lactation.^21^ It is well known that this prolactin expression pattern is synergistically regulated by dopamine, estrogen, thyroid-stimulating hormone, and several neuronal factors.^41^ Placental SOD3 depletion specifically inhibited the expression of prolactin but no other types of maternal behavior-related factors, such as oxytocin and estradiol. A possible reason is that regions outside the blood-brain barrier, such as the anterior pituitary and pineal gland, are markedly affected by extra-brain factors.^42^ Our data show that placenta-derived SOD3 is involved in extra-brain regulation of neuro-behavioral phenomena and prolactin expression during pregnancy.

Placental lactogen, a syncytiotrophoblast-derived hormone, is secreted into the maternal circulation, replacing the functions of pituitary prolactin during pregnancy.^20^ Although few studies have explored the placental origin of maternal mood disorders, clinical studies have reported that low levels of placental lactogens are associated with prenatal^43^ and postpartum^44^ depression. In rodents, placental lactogens are the main source of circulating lactogenic hormones in maternal blood from day 10 of pregnancy.^45^ Prolactin expression is rapidly increased by the disappearance of placental lactogen-induced negative feedback from day 18, and sucking maintains high levels of prolactin secretion throughout lactation. We found that placental *Sod3* KO attenuated the plasma levels of prolactin but not the expression levels of mouse placental lactogens (*Prl3b1*, *Prl3d1*, and *Prl3d3*). Considering the mechanism by which placental SOD3 epigenetically increases FGF/FGFR/prolactin signaling, placental SOD3 seems to boost prolactin induction at the late stage of pregnancy and nursing. However, our results do not rule out an important role in maternal behavior for placental lactogen. Recent studies have reported that the elimination of prolactin secretion during late pregnancy alone does not induce poor maternal behavior,^46^ suggesting compensative or additional roles of prolactin and placental lactogen in the regulation of maternal behavior.

One of our significant findings is that the effects of placental *Sod3* KO on maternal behavior are exhibited not only in F0 dams but also in F1 dams (Figure 6). This phenotype suggests that placental *Sod3* deletion results in both intergenerational and transgenerational effects on F1 and F2 offspring through prolactin dysregulation in F0 offspring. Similarly, a previous study reported that perinatally secreted maternal prolactin affects nurturing behaviors in adult offspring.^34^ Prolactin may function as a key regulator to establish maternal behavior through its generation. These types of intergenerational effects on offspring and grandoffspring have been also reported in maternal exercise^47^ and the microbiome.^48^ Since the placenta is developmentally derived from the embryo but histologically connected to both the mother and her embryo, it is difficult to separate the intergenerational effects from the transgenerational interaction in the placental contribution to F1 and F2. At least, we found that pregnant F1 offspring from placental *Sod3* KO dams have normal SOD3, prolactin, and oxytocin levels, indicating no change in the F2 epigenetic modifications of these hormone genes. Previous studies have reported that maternal behavior,^49,50^ maternal immune activation,^51^ maternal high-fat diet feeding-induced anxiety behavior,^52^ and postnatal social stress^53^ modulate maternal care and offspring behavior. Further investigation is needed to understand the transmission of impaired maternal behavior from the dam to offspring. However, placental SOD3 may be defined as one of the main regulators for the proper cultivation of maternal behavior through generations.

Maternal behavior is almost normal in WT conditions, and it is hard to show the significant effects of maternal exercise on maternal behavior without several types of intervention, such as maternal high-fat diet feeding, during pregnancy.^54–56^ However, maternal exercise significantly increased the levels of plasma prolactin (Figure 2E), pituitary prolactin (Figure 2F), FGFR2/FGF1 signaling axis (Figure 6A), DNA demethylation levels of Fgfr2 and Fgf1 (Figures 6G and 6I), and the expression of Zbtb18, a DNA demethylation inducer at the specific target genes (Figure 7). These results suggest that maternal exercise-induced placental SOD3 secretion potentially supports prolactin secretion via FGF/FGFR signaling.

In summary, our data demonstrate that placenta-derived SOD3 plays an important role in the establishment of maternal behavior during pregnancy. Mechanistically, SOD3 upregulates FGF1/FGFR2 signal induction through DNA demethylation at the promoters of these genes in the pituitary tissues of dams. Stimulatory factors of placental SOD3 secretion, such as exercise, may cultivate maternal behavior in dams. Additionally, sufficient production of placental SOD3 may contribute to maternal mood disorders during pregnancy and/or in the immediate postnatal period by inducing the FGF/FGFR axis in pituitary cells. Utilization of placental function may not only help pregnant mothers but also affect their children and subsequent generations.

### Limitations of the study

All mouse experiments were performed using only the C57BL/6 strain, and therefore, results cannot be generalized to other strains. Placental Sod3 deletion decreased prolactin levels via the increases of DNA methylation; however, the specific mechanisms underlying TET activation by SOD3 have not been determined. Although we observed the detrimental effects of Sod3 depletion on the maternal behavior of offspring, placental Sod3 expression was not affected in pregnancy offspring and, instead, implicates other unidentified factors in placental Sod3 KO-induced poor maternal behavior. Comprehensive omics analysis, including transcriptome and 5-mC/5-hmC DNA immnoprecipitation-seq, should be done to examine the global epigenetic changes in pituitary tissues from the dam. The detrimental effects of placental Sod3 KO on maternal behavior were partially recovered by embedding the osmotic pump with recombinant prolactin and SOD3. However, surgery experiments cannot continuously cover the placental Sod3 KO-induced prolactin impairment. For example, the epigenetic regulation cycle of the FGF1/FGFR2-prolactin axis is not fully restored by external prolactin supplementation. Finally, it is potentially important to determine whether daily exercise training would affect maternal behavior through placental SOD3.

## STAR★METHODS

### RESOURCE AVAILABILITY

#### Lead contact

Further information and requests for resources and reagents should be directed to and will be fulfilled by the lead contact, Joji Kusuyama (joji.kusuyama.bsin@tmd.ac.jp).

#### Materials availability

Mouse lines generated in this study are available from the lead contact upon request.

#### Data and code availability

- Datasets supporting the current study will be shared by the lead contact upon request.
- This paper does not report original code.
- Any additional information required to reanalyze the data reported in this paper is available from the lead contact upon request.

### EXPERIMENTAL MODEL AND SUBJECT DETAILS

#### Mouse models

Tpbpa/Ada Cre/*loxP* system was used to generate trophoblast-specific Sod3 knockout (*Sod3*^−/−^) and flox control (*Sod3*^f/f^) mice.^15^ Primer sequences used for genotyping are listed in Table S1. All animal studies were approved by the Animal Care and Use Committee of Tohoku University (Approved number: 2020–012-02) and conducted following the institutional guidelines. When we mated Sod3^f/f^ female mice with Tpbpa/Ada Cre^+/−^; Sod3^f/f^ male mice, offspring genotypes were theoretically hetero (HT: 50% of placentae in the dam are Sod3^f/f^ and 50% of placentae are Sod3^−/−^). In HT dam groups, we have confirmed all offspring genotypes (Cre^+^ = 50.89%/each dam). Our experiments only used HE dams after confirming 50% Cre^+^ offspring.

#### Primary cell culture

We modified the previous isolation methods for mouse primary pituitary cell cultures.^57,58^ After day 10 of delivery, the pituitary tissues of 10 to 12-week-old, WT or placental *Sod3*^−/−^ dams were immediately dissected, washed in PBS three times, and placed in the sterile cold α-MEM (137–17215, Fujifilm Wako) with 10% FBS, 10 nM HEPES, 50 units/mL penicillin, and 50 mg/mL streptomycin. Under sterile conditions, the anterior pituitaries were minced into small pieces by surgical blades and digested with 5 mL of α-MEM containing 0.1 mg/mL collagenase type II (LS004174, Worthington) and 0.15% trypsin (LS003702, Worthington) in 15 mL polypropylene tubes at 37°C with gentle and continuous rotation for 45 min. Enzymatic digestion was stopped by the addition of 5 mL of α-MEM with 10% FBS. The cell suspension was filtered through a 100 μm cell strainer to remove the debris and centrifuged at 150 × g for 5 min. After aspiration of supernatant, the cells were resuspended in α-MEM with 10% FBS, 50 units/mL penicillin, and 50 mg/mL streptomycin. 2 × 10^5^ cells were cultured on collagen-coated plates.

#### Cell line

GH3, a rat pituitary cell line, was obtained from JCRB Cell Bank (Osaka, Japan) and maintained in Ham’s F10 medium (087–08335, Fujifilm Wako) with 15% heat-inactivated horse serum, 2.5% fetal bovine serum (FBS), 50 units/mL penicillin, and 50 mg/mL streptomycin. The cells were cultured on collagen-coated plates.

### METHOD DETAILS

#### Breeding for maternal behavior experiments

8 to 10-week-old *Sod3*^*f/f*^ females were mated with 8 to 10-week-old *Sod3*^*f/f*^*; Tpbpa/Ada Cre*^−/−^ or *Sod3*^*f/f*^*; Tpbpa/Ada Cre*^*+/*−^ males. Upon induction of pregnancy, the dams were housed separately with a paper nestlet available for nest building. Nest building was scored from 0 to 4 (0: no nest, 1: flat, 2: cup, 3: incomplete dome, and 4: perfect nest with high walls).^59^ The number of pups born, number of living pups, number of pups with milk, and pup weight were assessed in the morning after birth. To evaluate the breastmilkfed pups, white milk spot (the milk-filled stomach) of the pups were observed through the transparent skin of the anterior abdominal wall. To evaluate the clean pups, the removed or attached placenta and extra-embryonic tissues were inspected in each neonatal pup. To assess the pups in the nest, the number of pups scattered in the cages and separated from each other by > 5 cm were counted and compared to the number of pups gathered in the nest area. Body weights of the pups were measured on 1–10 postnatal days. To isolate the breast milk, the mammary gland was excised and placed on a Petri dish on ice for 4h, and the excreted milk was collected.

#### Maternal behavior observation

Day of parturition is counted as Day 0. Maternal behavioral tests including retrieving, sniffing, grooming, and crouching were performed on postnatal day 1 (P1) after delivery. The dam was removed from the home cage for 10 min. Three pups were placed in the three corners of the home cage and the nest was placed in the fourth corner. The dams then returned to the corner of the nest facing the wall. The time of pup collection was monitored during a 1,000-s observation period by video recording. Latency to sniffing the first pup and retrieving the first pup to the nest was counted. Sniffing of pups was defined as the first nose contact of the dam with the pup. Retrieval of pups was defined as the dam picking up the pup from the corner and transporting it to the nest. Retrieval was scored only if the dam placed the pup entirely into the nest. Additionally, grooming (sniffing and licking the pups), and crouching (mother laying in a nursing posture on top of the pups, and at least two collected pups under the ventral side of the body) were recorded.

#### Exercise training program by wheel running

8 to 10-week-old female *Sod3*^*f/f*^ mice were fed chow diet (Labo MR Stock, Nosan corporation) for 2 weeks preconception, during gestation, and until pup weaning. Mice were additionally divided into two subgroups: trained (mice housed with running wheel preconception and during gestation) and sedentary (mice housed in static cages). Male breeders were 10 to 12-week-old *Sod3*^*f/f*^*; Tpbpa/Ada Cre*^*+/*−^ sedentary mice that were maintained on a chow diet. To control for potential differences between the sires, breeding was performed as harems.

#### Reverse transcription-quantitative PCR (RT-qPCR)

Total RNA was isolated with Isogen II (311–07361, Nippon Gene), and reverse transcribed with iScript Reverse Transcription Supermix for RT-qPCR (1708841, Bio-Rad). Complementary DNA was amplified with Fast SYBR Green Master Mix (4385617, Applied Biosystems) using the StepOnePlus Real-Time PCR System (4376598, Applied Biosystems). For each gene, the mRNA expression was calculated relative to that of Rpl13a. Primer sequences used for RT-qPCR analysis of mouse and rat samples are listed in Table S2.

#### Prolactin and SOD3 treatment via osmotic pump infusion *in vivo*

8 to 10-week-old female *Sod3*^*f/f*^ mice were mated with 8 to 10-week-old male *Sod3*^*f/f*^*; Tpbpa/Ada Cre*^*+/+*^ mice, maintained on a chow diet, and were sedentary during gestation. Then, 13.5 days post coitus (dpc), the dams underwent surgery to have osmotic pumps (2001 [nominal pumping rate: 1.0 μL/h, nominal duration: 1-week, nominal reservoir volume 200 μL], Alzet) implanted adjacent to the subcutaneous fat pad. Osmotic pumps were filled with 7.5 μg of recombinant mouse prolactin (1445-PL, R&D Systems) or SOD3 diluted in 1 mL of phosphate-buffered saline (PBS). PBS-filled pumps were implanted into the control groups. During 7 consecutive days, the mice received 10 ng of prolactin or recombinant SOD3^15^ per gram of body mass per hour. Maternal behavioral tests were performed after delivery.

#### Cabergoline injection *in vivo*

8 to 10-week-old female *Sod3*^*f/f*^ mice were mated with 8 to 10-week-old male Sod3f/f; Tpbpa/Ada Cre+/+ mice, maintained on a chow diet, and were sedentary during gestation. The dams were intraperitoneally injected with 0.1 mg/kg/day of cabergoline (23934, Cayman) or saline from day 12.5–18.5 of pregnancy. Maternal behavioral tests were performed after delivery.

#### Recombinant proteins and inhibitors

The human recombinant SOD3 protein was produced as previously described.^16^ Recombinant mouse FGF1 (4686-FA, R&D Systems), FGF2 (062–05181, Fujifilm Wako), and FGF4 (5846-F4, R&D Systems) were obtained commercially. BGJ398 (19157; FGFR1, 2, 3 inhibitor), PD166866 (22464; FGFR1 inhibitor), and H3B-6527 (26072; FGFR4 inhibitor) were obtained from Cayman Chemical.

#### RNA sequencing

Samples were quantified with an Agilent 4200 Tapestation instrument, using a corresponding Agilent High Sensitivity RNA assay. The resulting RNA Integrity Number (RIN) scores and concentrations were considered to qualify the samples for further analysis. Poly (A) RNA was prepared using Poly (A) mRNA Magnetic Isolation Module (E7490, NEB). Library preparation was performed using the NEBNext Ultra II Directional RNA Library Prep Kit for Illumina (E7760, NEB). The pool was denatured and loaded onto a NovaSeq 6000 (Illumina) using an Illumina NextSeq High Output 150-cycle kit to obtain Paired-End 75bp reads. The pool was loaded at 1.9 p.m., with 5% PhiX spiked in to serve as a sequencing control. The resulting FASTQ files were used for subsequent analysis. Bioinformatics analysis was conducted as previously described.^15^

#### Western blotting

Lysates were analyzed as previously described.^15^ Primary antibodies against FGF1 (ab207321, abcam), FGFR2 (SAM4500889, Sigma-Aldrich), phospho-PI3K (17366, CST), phospho-PKCδ (2055, CST), PKCδ (2058, CST), phospho-PLCγ1 (14008, CST), PLCγ1 (5385, CST), and βTubulin (2128, CST) were commercially obtained.

#### Methylation-specific PCR (MSP)

MSP was performed as previously described.^15^ Quantitative MSP was performed at the pair of methylation primers (M-primers) and unmethylation primers (U-primers) targeting each promoter region. M-primers and U-primers were designed using the Methyl Primer Express Software (Applied Biosystems). All primer sequences used in PCR are listed in Table S3.

#### 5-hmC DNA immunoprecipitation qPCR (5-hmC DIP-qPCR)

5-hmC DIP-qPCR was conducted as previously described.^15^ All primer sequences used in PCR are U Primer which listed in Table S3.

#### Enzyme-linked immunosorbent assay (ELISA) and biochemical assays

Serum, plasma, and pituitary tissue levels of SOD3 (OKCD01107, Aviva System Biology), prolactin (ab100736, abcam), oxytocin (292–84401, Fujifilm Wako), estradiol (KGE014, R&D Systems), progesterone (CSB-E05104m, Cusabio), corticosterone (ab108821, abcam), dopamine (BA E–5300R, ImmuSmol), placental lactogen (LS-F28728–1, LifeSpan BioSciences), FGF1 (DY4686–05, R&D Systems), FGF2 (DY3139–05, R&D Systems), and FGF4 (ELM-FGF4–1, Ray Biotech) were determined using ELISA according to the manufacturer’s instructions. Tissue levels of αKG were determined using an alpha KG Assay Kit (ab83431, abcam). IDH activity was analyzed by the Isocitrate Dehydrogenase Assay Kit (ab102528, abcam). TET activity was analyzed using the TET Hydroxylase Activity Quantification Kit (ab156913, abcam). The sensitivity and itra-assay and inter-assay coefficient of variations were shown in Table S4.

#### Mating system to analyze the maternal behavior of the offspring

To analyze the effects of *Sod3* depletion on the maternal behavior of the offspring (F1), four experimental groups were set by the combination of dams, sires, foster mothers, and offspring male partner genotypes (Figures 6A and 6B). First, 8 to 10-week-old female (F0) were mated with 8 to 10-week-old males. The offspring at E18.5 were removed from the abdomen, and the resuscitated newborns were parented by the foster mother from birth onwards. Next, 8-week-old female offspring were mated with 8 to 10-week-old male. Offspring behavioral tests were performed on postnatal day 1 after the delivery of the grand-offspring (F2).

### QUANTIFICATION AND STATISTICAL ANALYSIS

The dots represent the different dams or cells in all figures. All the dams were derived from different mothers and housed in separate cages. All experiments were conducted simultaneously. This study used at least three replicates for each observation dimension unless otherwise stated. For animal experiments, five to eight replicates were set for each observation dimension.

All data are represented as the means ± SEM. Statistical significance was defined as *p* < 0.05, 0.01, 0.001, or 0.0001 and determined via one- or two-way ANOVA with Tukey and Bonferroni post hoc analysis. For experiments conducted at various ages, statistical analyses were performed based on the control group at each time point and no comparisons among ages were performed.

## Supplementary Material

1

## Figures and Tables

**KEY RESOURCES TABLE:** 

REAGENT or RESOURCE	SOURCE	IDENTIFIER

Antibodies		

FGF1	abcam	Cat# ab9588; RRID:AB_308729
FGFR2	Sigma-Aldrich	Cat# SAB4500889
phospho-PI3K	Cell Signaling Technology	Cat# 17366; RRID:AB_2895293
phospho-PKCδ	Cell Signaling Technology	Cat# 2055; RRID:AB_330876
PKCδ	Cell Signaling Technology	Cat# 2058; RRID:AB_10694655
phospho-PLCγ1	Cell Signaling Technology	Cat# 14008; RRID:AB_2728690
PLCγ1	Cell Signaling Technology	Cat# 2822; RRID:AB_2163702
βTubulin	Cell Signaling Technology	Cat# 2128; RRID:AB_823664
5-hmC antibody	Active Motif	Cat# 39769; RRID:AB_10013602

Chemicals, peptides, and recombinant proteins		

Recombinant Mouse Prolactin Protein	R&D Systems	1445-PL
Recombinant Mouse SOD3 Protein	Kusuyama et al.^15^	N/A
Cabergoline	Cayman Chemical	23934
Recombinant Mouse FGF1 Protein	R&D Systems	4686-FA
Recombinant Mouse FGF2 Protein	Fujifilm Wako	062–05181
Recombinant Mouse FGF4 Protein	R&D Systems	5846-F4
BGJ398	Cayman Chemical	19157
PD166866	Cayman Chemical	22464
H3B-6527	Cayman Chemical	26072

Critical commercial assays		

SOD3 ELISA Kit (Mouse)	Aviva System Biology	OKCD01107
Mouse Prolactin ELISA Kit	abcam	ab100736
Oxytocin ELISA Kit Wako	Fujifilm Wako	292-84401
Estradiol Parameter Assay Kit	R&D Systems	KGE014
Mouse progesterone (PROG) ELISA Kit	Cusabio	CSB-E05104m
Corticosterone ELISA kit	abcam	ab108821
Dopamine ELISA kit – Fast Plasma & Urine samples	ImmuSmol	BA E–5300R
Mouse Placental Lactogen (Sandwich ELISA) ELISA Kit	LifeSpan BioSciences	LS-F28728-1
Mouse FGF acidic/FGF1 DuoSet ELISA	R&D Systems	DY4686-05
Mouse FGF basic/FGF2/bFGF DuoSet ELISA	R&D Systems	DY3139-05
Mouse FGF-4 ELISA Kit	Ray Biotech	ELM-FGF4-1
Alpha Ketoglutarate (alpha KG) Assay Kit	abcam	ab83431
Isocitrate Dehydrogenase Assay Kit (Colorimetric)	abcam	ab102528
TET Hydroxylase Activity Quantification Kit (Fluorometric)	abcam	ab156913

Experimental models: Cell lines		

GH3	JCRB Cell Bank	JCRB9047

Experimental models: Organisms/strains		

Sod3^f/f^ mice	Kusuyama et al.^15^	N/A
Tpbpa/Ada Cre mice	Kusuyama et al.^15^	N/A
Tpbpa/Ada Cre; Sod3^f/f^ mice	This paper	N/A
C57BL/6	Charles River	M/A

Software and algorithms		

Prism 10	GraphPad	N/A
Methyl Primer Express	Applied Biosystems	N/A

Other		

Chow diet	Nosan corporation	Labo MR Stock
Wheel running cage	MELQUEST	RWC-15
Osmotic pump	Alzet	2001

## Highlights

- Placenta-specific *Sod3*^−/−^ dams exhibited impaired maternal behavior and prolactin secretion
- FGF pathways and PI3K-PLCγ1-PKCδ axis were downregulated in pituitary gland of *Sod3*^−/−^ dams
- SOD3 inhibits TET-induced DNA demethylation at the ZBTB18-targeted promoters of Fgf1/Fgfr2
- Offspring from *Sod3*^−/−^ dams showed impaired nurturing behavior to their gland offspring
