# Supplementary material for: Placenta-derived SOD3 deletion impairs maternal behavior via alterations in FGF/FGFR-prolactin signaling axis
Source: Cell Rep. Author manuscript; Available in PMC 2024 Dec 13. (PMC11639441; doi:10.1016/j.celrep.2024.114789)
Supplement: 1 [file NIHMS2038426-supplement-1.pdf]

Cell Reports, Volume *43*

Supplemental information

Placenta-derived SOD3 deletion impairs maternal behavior via alterations in FGF/FGFR-prolactin signaling axis

Yidan Xu, Ana B. Alves-Wagner, Hitoshi Inada, Sepideh D. Firouzjah, Shion Osana, Muhammad Subhan Amir, Royce H. Conlin, Michael F. Hirshman, Eva S. Nozik, Laurie J. Goodyear, Ryoichi Nagatomi, and Joji Kusuyama

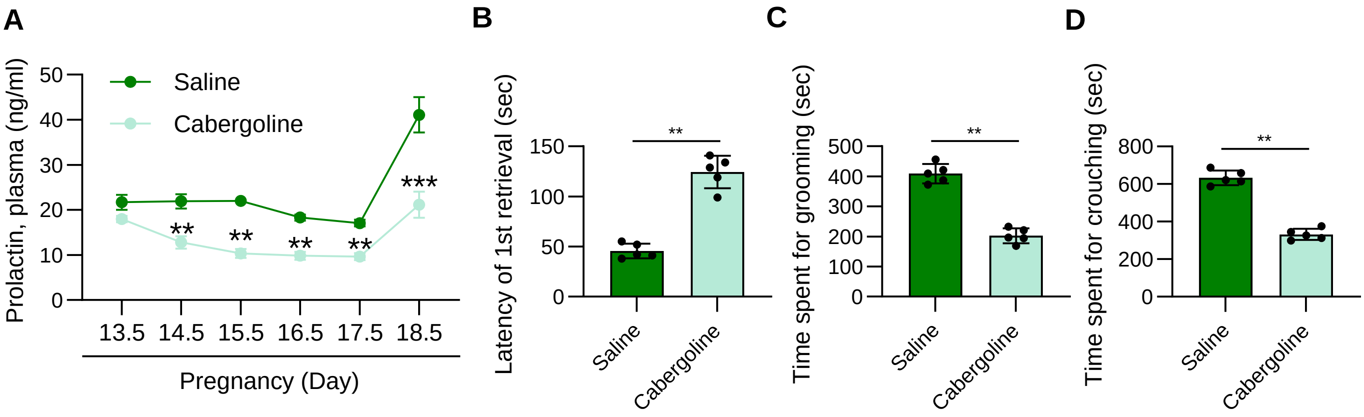

Supplemental Figure 1: Cabergoline injection inhibits prolactin levels and maternal behavior, Related to Figure 4. A) Plasma levels of prolactin of daily cabergoline injected dams from day 13.5 to 18.5. B-D) Latency of the first retrieval on day 1 (B), time spent for grooming on day 3 (C), and time spent for crouching on day 1 (D) of daily cabergoline injected dams. (N=5 in each group. Three technical replicates for each group. **P<0.01, ***P<0.001)

Supplemental Table 1: List of primer sequences used for genotyping, Related to Figure 1.

Gene	Forward	Reverse
Sod3 flox	GATGACCTGGGAACATGATGGAGAGG	GCCTGTTCTGCTAAGCTCTCACAAAC
Tpbpa/Ada Cre	CGGTCTCTGAGAGCCATC	CCCTGAACATGTCCATCA
Tpbpa/Ada Cre for qPCR	TTTCCATATTGCAGAACGAAAACG	CAGGCTAAGTGCCTTCTCTACA
Internal control for qPCR	TTTAAGTGGCTTGCCATTTCTGG	TCATCCTACAGTGGAAGGATTCAC

Supplemental Table 2: List of primer sequences used for RT-qPCR of mouse and rat samples, Related to Figure 2, 4, 5, 6, 7

Gene	Description	Forward	Reverse
mAvp	Arginine vasopressin	GCTACTTCCAGAACTGCCCAAG	CAGCAGATGCTTGGTCCGAAGC
mAvpr1b	Arginine vasopressin receptor 1B	GGCACCTTTCTTCAGTGTCCAG	GTTGCCCAGAAGCATCGAGATG
mDrd2	Dopamine receptor D2	CCTGTCCTTCACCATCTCTTGC	TAGACCAGCAGGGTGACGATGA
mFgf1	Fibroblast growth factor 1	CCAAGGAAACGTCCACAGTCAG	ACGGCTGAAGACATCCTGTCTC
mFgf2	Fibroblast growth factor 2	AAGCGGCTCTACTGCAAGAACG	CCTTGATAGACACAACTCCTCTC
mFgfr1	Fibroblast growth factor receptor 1	GCCTCACATTCAGTGGCTGAAG	AGCACCTCCATTTCCTTGTCGG
mFgfr2	Fibroblast growth factor receptor 2	GTCTCCGAGTATGAGTTGCCAG	CCACTGCTTCAGCCATGACTAC
mFgfr3	Fibroblast growth factor receptor 3	ACAGGTGGTCATGGCAGAAGCT	CTCCATCTCAGATACCAGGTCC
mFgfr4	Fibroblast growth factor receptor 4	TCCGACAAGGATTTGGCAGACC	TGGCGGCACATTCCACAATCAC
mIdh1	Isocitrate dehydrogenase 1	CAGGCTCATAGATGACATGGTGG	CACTGGTCATCATGCCAAGGGA
mIdh2	Isocitrate dehydrogenase 2	GGCTGTCAAGTGTGCCACAATC	TTGGCTCTCTGAAGACGGTTCC
mOxt	Oxytocin	CTGTGCTGGACCTGGATATGCG	AGCTCGTCCGCGCAGCAGATG
mOxtr	Oxytocin receptor	TCATCGTGTGCTGGACGCCTTT	GCCCGTGAAGAGCATGTAGATC
mPrl	Prolactin	CTGGCTACACCTGAAGACAAGG	TCACTCGAGGACTGCACCAAAC
mPrlr	Prolactin receptor	GACCTGCATCTTTCCACCAGTTC	AGAACTCCACCAGCAAGTCCTC
mPrl3b1	Prolactin family 3, subfamily b, member 1	AAACAGCGAGCAAGTCCACCAG	TGCCACCATGTGTTTCAGAGGC
mPrl3d1	Prolactin family 3, subfamily d, member 1	CCAGAGAATCGAGAGGAAGTCC	GTCAGTGCAGACACCAGGTGTT
mPrl3d3	Prolactin family 3, subfamily d, member 3	CCAGAGAATCGAGAGGAAGTCC	TCCTGGAAGAGCAGTCAGTGCA
mSod3	Superoxide dismutase 3	GACCTGGTTGAGAAGATAGGCG	TGGCTGATGGTTGTACCCTGCA
mTet1	Ten-eleven translocation methylcytosine dioxygenase 1	CAGGACCAAGTGTTGCTGCTGT	GACACCCATGAGAGCTTTTCCC
mTet2	Ten-eleven translocation methylcytosine dioxygenase 2	ACCTGGCTACTGTCATTGCTCC	TGCAGTGACTCCTGAGAATGGC
mTh	Tyrosine hydroxylase	TGCACACAGTACATCCGTCATGC	GCAAATGTGCGGTCAGCCAACA
mZbtb18	Zinc finger and BTB domain containing 18	CTGTCAAGTCCAGCCTTTCAGG	CTCATCACAGGACGCCTCTTTC
rPrl	Prolactin	GTCAGCCCGGAAAGCAGG	ACCACACGGTCAAACAGCTC
rPrlr	Prolactin receptor	AATCATCCAGAGAGGGGCTC	ATCAGAGGCTCCCTTCACAG
rRpl13a	Ribosomal protein L13a	GCGGAGGGGCAGGTTCTA	CTCACAGCGTACAACCACCA

Supplemental Table 3: List of primer sequences used for MSP, Related to Figure 6, 7.

	Target site		Forward	Reverse
Fgfr1	-308 to -143	M Primer	GGAAAATCGAGATTTAGAGTAC	ACGCCCGAATTTCAAACTAT
		U Primer	GGGAAAATTGAGATTTAGAGTAT	AACACCCAAATTTCAAACTAT
Fgfr2	-245 to -90	M Primer	GTGTATTTTTGCGGGTAGC	CCGAACCGTTTCCTAAAC
		U Primer	TTTGTGTATTTTTGTGGGTAGT	ACCCAAACCATTTCCTAAACA
Fgfr3	-150 to -64	M Primer	TCGGTATTGTTTTAAGGGGTC	CGAACGATCCCAAAAAAC
		U Primer	AGTTTGGTATTGTTTTAAGGGGTT	CCAAACAATCCCAAAAAACAT
Fgfr4	-159 to -16	M Primer	ACGTGGGGTTTCGGAAC	TCCGAAACCCGACAAATATC
		U Primer	TGGATGTGGGGTTTTGGAAT	TCCAAAACCCAACAAATATCAAC
Fgf1	-933 to -695	M Primer	TTGGGATATATACGAATACGTATAC	CATAATTAATTATTTCCGATCG
		U Primer	AGGTTGGGATATATATGAATATGTATAT	AACCATAATTAATTATTTCCAATCA
Fgf2	-154 to -72	M Primer	AAATTTAGGAGTTGCGTTATC	CCGACGCCTCCAAATTAC
		U Primer	TTTAAATTTTAGGAGTTGTGTTATT	CCCAACACCTCCAAATTACAA
Ptp4a1	-231 to -105	M Primer	AGGCGTTTTAAGTTGAGGC	CAAACGTCACTCGAAACAAC
		U Primer	GAGAGGTGTTTTAAGTTGAGGT	ACACAAACATCACTCAAAACAAC
Gng8	-685 to -545	M Primer	AAATTAGTTGCGGGAGGC	TAAAAAATATCAAACGCGCC
		U Primer	TGAAAATTAGTTGTGGGAGGT	CTAAAAAATATCAAACACACCAA
Hspa1b	-304 to -136	M Primer	TTTATAGGGATTTCGAAGTTGC	CGCCTCCCTTAAATAATCG
		U Primer	TTATTTATAGGGATTTTGAAGTTGT	CCCCACCTCCCTTAAATAATCA
Crabp2	-321 to -202	M Primer	TGAAACGGGATTTGTGGTTC	GAACCGAAAAACGTCAAAAA
		U Primer	TGGTGAAATGGGATTTGTGTTT	AAACCAAAAAACATCAAAAAACA
Cyp26b1	-189 to -44	M Primer	AGAGTCGAGAGATTGCGTTTC	AAAACCCCGAACGAACTCT
		U Primer	TTTAGAGTTGAGAGATTGTGTTTT	AACAAAACCCCAAACAAACTCT
Rab43	-503 to -362	M Primer	TAGGGTTACGAAAAGGTGC	TACGAATTCGCTATCAACAAC
		U Primer	TTTTAGGGTTATGAAAAGGTGT	TACAAATTCACTATCAACAACACC

Supplemental Table 4: ELISA information, Related to Figure 2, 3, 4, 6, 8.

Target	Sensitivity	Intra-assay and inter-assay coefficient of variations (CVs) (%)
SOD3	1.42 ng/mL	4.9
Prolactin	30 pg/mL	3.9
Oxytocin	4 pg/mL	5.2
Estradiol	12.1 pg/mL	4.8
Progesterone	0.3 ng/mL	6.3
Corticosterone	0.28 ng/mL	6.1
Dopamine	25 pg/ml	5.1
Placental lactogen	1 ng/mL	8.9
FGF1	12.5 pg/mL	4.5
FGF2	46.9 pg/mL	5.2
FGF4	No information	5.3
Alpha KG	0.01 nmol/well	4.8
IDH activity	0.01 mU/well	6.1
TET activity	10 ng/well	5.3
